# How Human Tumor Viruses Make Use of Autophagy

**DOI:** 10.3390/cells1030617

**Published:** 2012-08-27

**Authors:** Zachary L. Pratt, Bill Sugden

**Affiliations:** 1 Department of Bacteriology and Food Research Institute, Microbial Sciences Building, University of Wisconsin-Madison, Madison, WI 53706, USA; Email: zpratt@wisc.edu; 2 Department of Oncology, McArdle Laboratory for Cancer Research, Madison, WI 53706, USA

**Keywords:** Epstein-Barr Virus, Kaposi’s Sarcoma Herpesvirus, Hepatitis B Virus, Hepatitis C Virus, human tumor virus, autophagy, cancer

## Abstract

Viruses commandeer regulatory pathways of their hosts to optimize their success as cellular parasites. The human tumor viruses, Epstein-Barr Virus (EBV), Kaposi’s Sarcoma Herpesvirus (KSHV), Hepatitis B Virus (HBV), and Hepatitis C Virus (HCV) all affect autophagy for their own ends. EBV and KSHV regulate it during latent infections, a phase when no progeny virus is produced, while HBV and HCV use autophagy to promote their productive infections. Here we shall compare and contrast how these human tumor viruses regulate autophagy and what they gain by the appropriation of this cellular pathway.

## 1. Introduction

Autophagy is a cellular program that can be initiated by several stimuli, is carried out by multiple regulatory molecules and enzymes, and culminates in the formation of double-membrane bound vesicles, termed autophagosomes, containing macromolecules which are catabolized in autophagolysosomes upon fusion of autophagosomes with lysosomes [1,2]. To initiate autophagy, the phosphatidylinositol 3-phosphate kinase, Vps34 (abbreviations can be found in Table 1), must be activated and generate phosphatidylinositol 3-phosphate for the growing phagophore, a precursor structure to the autophagosome. The stimuli for autophagy range from nutrient starvation to acute exercise [3]. The autophagic program is controlled by regulators as diverse as mammalian target of rapamycin (mTOR) and B-cell chronic lymphocytic leukemia/lymphoma 2 (Bcl2) [1] and the participants in it include kinases, ubiquitin-like conjugating molecules, phosphatidylinositol 3-kinases, and transmembrane proteins [4]. 

**Table 1:** Full names of abbreviations used in this review.

Abbreviation	Full Name
3-MA	3-methyl adenine
6TM	6-transmembrane-spanning domain of LMP1
AIDS	acute immunodeficiency syndrome
ATF	activated transcription factor
ATG	autophagy-related
BALF1	*Bam*HI A leftward fragment 1
Bcl2	B-cell chronic lymphocytic leukemia/lymphoma 2
BHRF1	*Bam*HI H rightward fragment 1
CD	cluster of differentiation
CRE	cAMP responsive element
EBNA1	Epstein-Barr nuclear antigen 1
EBV	Epstein-Barr Virus
eIF2α	eukaryotic initiation factor 2 alpha
HBs	Hepatitis B small surface protein
HBV	Hepatitis B Virus
HBx	Hepatitis B X protein
HCC	hepatocellular carcinoma
HCV	Hepatitis C Virus
HFFs	human foreskin fibroblasts
IFN	interferon
IRGM	immunity related GTPase M
ISG56	interferon-stimulated gene 56
KSHV	Kaposi's Sarcoma Herpesvirus
LC3	light chain 3
LMP1	latent membrane protein 1
MAVS	mitochondrial antiviral signaling protein
MHC	major histocompatibility complex
mTOR	mammalian target of rapamycin
NAF1	nutrient-deprivation autophagy factor 1
NFkB	nuclear factor kappa B
NS4B	non-structural protein 4B
PE	phosphatidylethanolamine
PEL	primary effusion lymphoma
PERK	protein kinase RNA-like endoplasmic reticulum kinase
RIG-I	retinoic acid inducible gene I protein
RTA	replication and transcription activator
siRNA	small interfering RNA
TNFα	tumor necrosis factor alpha
UPR	Unfolded Protein Response
v-FLIP	FADD-like interleukin-1 beta-converting enzyme inhibitory protein
Vps34	vesicle-mediated vacuolar protein sorting 34

Viruses are obligate cellular parasites and human tumor viruses, in particular, are obligate cellular parasites that can affect their host cells oncogenically. Viruses need to amplify their numbers to survive and spread. They use the synthetic machinery of their host cells to make more of themselves and these productive cycles often end in cell death and pathology. Human tumor viruses must produce more of themselves, too, but can drive the infected cells both to survive and proliferate aberrantly. Several human tumor viruses make use of autophagy either during their productive cycles, or during non-productive infections, or both (Table 2). Here we review how four human tumor viruses involve autophagy in their life-cycles to foster their own parasitic ends and to induce their pathologies. 

**Table 2:** Multiple roles for autophagy in the life-cycles of human tumor viruses.

Virus	Role of Autophagy in Viral Life-cycle	References
EBV	Autophagy contributes to the generation of epitopes of EBNA1, a pivotal viral protein, presented on MHC II	[5,6]
	Along with the UPR, autophagy regulates expression of LMP1, a viral oncogene	[7,8]
KSHV	Autophagy inhibits survival and proliferation of latently-infected cells Supports viral replication and productive life-cycle	[9,10]
		[11]
HCV	Autophagy blocks an interferon response during the early stages of infection allowing HCV to establish its infection	[12]
HBV	Autophagy supports infection and may provide a scaffold for reverse transcription or viral envelopment during the productive life-cycle	[13,14]

## 2. Epstein-Barr Virus

Epstein-Barr Virus (EBV) is a Herpesvirus, and as with other members of this family, its viral particle is enveloped and contains a double-stranded DNA genome of 165 kbp. EBV infects the vast majority of people in the world without causing disease, but does cause a variety of cancers in a minority of infected people including Burkitt’s lymphoma, Hodgkin’s lymphomas, B-cell post-transplant lymphoproliferative disorder, Nasopharygeal carcinoma, and gastric carcinoma [15]. Once people are infected, the virus is latently maintained in memory B-cells for the life of the person [16]. There are at least two apparently unrelated ways in which EBV has evolved to use autophagy in its host cell. EBV DNA is maintained as an extrachromosomal plasmid in latently infected cells, including all tumor cells. The virus contributes only one protein, Epstein-Barr nuclear antigen 1 (EBNA1), to the synthesis and maintenance of its plasmid; all other *trans*-acting factors are provided by the host cell [17]. EBNA1, though, is essential for EBV to be maintained in proliferating cells [18,19]. 

Surprisingly, the expression of EBNA1 in infected cells does not lead to those cells being recognized efficiently by cluster of differentiation 8 (CD8)-positive cytotoxic T-cells. A stretch of glycine-alanine repeats in this viral protein limits its presentation as an antigen on major histocompatibility complex (MHC) class I molecules [20]. Rather, EBNA1 stimulates CD4-positive T-cells when presented on infected B-cells by MHC class II molecules [5,21,22]. Some of these epitopes are generated via autophagy, and EBNA1 can be found in double membrane vesicles, consistent with its localization to autophagosomes (Table 2) [6,23,24]. Stimulation of CD4-positive T-cells after their exposure to EBV-positive cells is inhibited when autophagy is suppressed with small interfering RNAs (siRNAs) that target mediators of autophagy, ATG12 or ATG7 (ATG refers to “autophagy related”) [6,23]. Similarly, CD4-positive T-cells remain unstimulated when the acidification of autophagolysosomes in infected cells is inhibited [23]. Despite its immunogenicity, EBNA1 is poorly presented on MHC II molecules, perhaps because it spends little time in the cytoplasm [5,6,25,26]. By disrupting its nuclear localization signal and rendering it cytosolic, Leung and colleagues were able to enhance EBNA1’s degradation in autophagolysosomes and increase the stimulation of CD4-positive T-cell clones by 15-fold [6]. How the presentation of EBNA1 to CD4-positive T-cells contributes to EBV’s life-cycle is unclear. However, its poor presentation by MHC class II molecules surely contributes to some EBV-positive tumors, such as canonical Burkitt’s lymphomas, in which EBNA1 is the only viral protein expressed, escaping the hosts’ immune response.

There is potentially a mechanism by which EBV regulates autophagy to affect the host’s immune response. EBV encodes two proteins, *Bam*HI rightward fragment 1 (BHRF1) and *Bam*HI A leftward fragment 1 (BALF1), which are orthologs of the cellular antiapoptotic protein, Bcl2. These proteins can each inhibit apoptosis and at least one or the other needs to be expressed in newly infected B-lymphocytes for EBV to infect the cells successfully [27]. BHRF1 is expressed in a subset of Burkitt’s lymphomas and helps to block apoptosis in these latently infected tumor cells, too [28]. Bcl2 and its ortholog encoded by KSHV bind Beclin1 and thereby inhibit autophagy [29]. If the EBV-encoded orthologs of Bcl2, BHRF1 and BALF1, bind Beclin1 too, which appears likely, then these EBV proteins will also inhibit autophagy. Their potential inhibition of autophagy would limit further the presentation of EBNA1-derived peptides by MHC class II molecules and simultaneously limit any other contributions to innate immunity mediated through autophagy. 

EBV uses autophagy to regulate expression of one of its oncogenes, the latent membrane protein 1 (LMP1), which drives proliferation of infected B-cells (Table 1). LMP1 is expressed in a subset of latent infections and contains two domains that perform independent functions [30]. Its carboxy-terminal domain is a viral mimic of the human CD40 receptor, and activates signaling pathways, such as nuclear factor kappa B (NFkB), to drive cellular proliferation and inhibit apoptosis [7,30,31,32]. This carboxy-terminal signaling requires LMP1 to self-oligomerize, which is accomplished by a second domain, its six-transmembrane spanning domains (6TM) [8]. Recently, it has been shown that a single amino acid within transmembrane domain 5 is necessary for LMP1’s oligomerization [33]. The 6TM of LMP1 dose-dependently induces autophagy via the Unfolded Protein Response (UPR) (Table 3) [34,35]. This induction is mediated at least in part by LMP1’s activating protein kinase RNA-like endoplasmic reticulum kinase (PERK), a kinase that can phosphorylate eukaryotic initiation factor 2 alpha (eIF2α). These cellular mechanisms are used to regulate LMP1’s own synthesis and degradation, thereby modulating its expression and signaling during infection (Table 2). When levels of LMP1 within B-cells are low, LMP1 promotes its own transcription by activating PERK to phosphorylate eIF2α [34,36]. An activated transcription factor/ cAMP responsive element (ATF/CRE) element in LMP1’s promoter is activated during the UPR, likely via ATF4 whose translation is enhanced by phospho-eIF2α [35]. Translation of LMP1 is mediated by unphosphorylated eIF2α, which makes up the majority of the total eIF2α in these cells [35]. However, as the levels of LMP1 increase, the fraction of eIF2α phosphorylated increases and translation of LMP1 is limited [35]. 

**Table 3:** Viral proteins that affect autophagy.

Virus	Viral Protein	Autophagic Protein	Effect of Viral Proteins on Autophagy	References
EBV	LMP1	Unknown	Activates autophagy via the UPR	[7,8]
KSHV	v-FLIP	ATG3	Competes with LC3 for binding to ATG3, thereby inhibits the conjugation of LC3 to PE	[10]
	v-Bcl2	Beclin 1	Binds Beclin 1 and inhibits its activation of autophagy	[29,37,38]
	RTA	Unknown	Induces autophagy	[11]
HCV	NS4B	Vps34	Induces autophagy by stimulating the enzymatic activity of Vps34	[39]
	NS5B	ATG5	Stimulates conjugation of LC3 to PE	[40]
HBV	HBs	Unknown	Activates autophagy via UPR	[41]
	HBx	Vps34	Initiates autophagy, possibly by the generation of phosphatidylinositol 3-phosphate by Vps34	[14]

To induce its degradation, LMP1 stimulates autophagy via the UPR [34]. LMP1’s activation of PERK likely contributes to the activation of autophagy via PERK’s phosphorylation of eIF2α. The transcription factor, ATF4, which is translated by phospho-eIF2α, can stimulate transcription of genes encoding the autophagic proteins, light chain 3 (LC3) and autophagy-related 5 homolog (ATG5) [42]. LC3 is an ubiquitin-like protein that is conjugated to phosphatidylethanolamine (PE) [4]. LC3 is translated as an inactive protein (Pro-LC3), which is cleaved by the protease, ATG4, to generate cytosolic LC3-I. LC3-I is conjugated to PE (LC3-II) in a reaction requiring the E2-like enzyme, ATG3, and the E3-like complex, ATG16L complex [4]. The lipid-conjugation of LC3 allows LC3’s insertion into the phagophore. Inhibiting autophagy or the acidification of lysosomes blocks LMP1’s degradation, thus increasing its expression in EBV-positive cells [10,34]. Disrupting this complex regulation of expression is deleterious to the cell and inhibits cellular survival and/or proliferation. The levels of LMP1 vary in otherwise genetically identical cells; those expressing intermediate levels of LMP1 proliferate optimally, while those with low or high levels grow more slowly and likely will avoid immune detection [9,36]. A weak immune response will be elicited if too little antigen is expressed. When expressed at high levels, LMP1’s 6TM inhibits proliferation, thereby rendering B-cells resistant to external apoptotic stimuli, such as tumor necrosis factor alpha (TNFα) [9]. This complex regulation of the LMP1 oncogene contributes to EBV’s driving proliferation of both normal and some tumor cells. 

## 3. Kaposi’s Sarcoma Herpesvirus

Kaposi’s Sarcoma Herpes Virus (KSHV) is also a Herpesvirus having an enveloped virus particle encapsidating a double-stranded DNA genome. The prevalence of infection by KSHV varies dramatically in different populations. Between 5%–10% of adult North Americans are infected on average, while up to 65% of isolated groups of indigenous people in Brazil are infected [43]. KSHV causes Kaposi’s Sarcoma, an endothelial cancer associated often with acute immunodeficiency syndrome (AIDS), and primary effusion lymphomas (PELs), a lymphoma of B-cell origin [43]. Most PEL cells are infected both with KSHV and EBV [43]. PEL cells do not usually express LMP1, and therefore autophagy does not affect expression of this viral oncogene. 

KSHV appears to have a love/hate relationship with autophagy. It blocks autophagy during the latent phase of its life-cycle and depends on it during the productive phase of its life-cycle. KSHV encodes FADD-like interleukin-1 beta-converting enzyme inhibitory protein (v-FLIP), which inhibits autophagy [44]. v-FLIP does so by competing with LC3 for binding to ATG3, thereby inhibiting LC3’s conjugation to PE and its insertion into phagophores (Table 3) [4,44]. The effect of v-FLIP on autophagy is at least two-fold (Table 2). First, v-FLIP inhibits autophagic cellular death [44]. Lee and colleagues examined how inhibiting v-FLIP’s binding to ATG3 would affect tumor development by PEL cells grown as xenografts. They developed peptide mimics of the α-2 and α-4 domains of v-FLIP, which compete specifically for v-FLIP’s binding to ATG3, and injected them into mice receiving KSHV-positive xenografts. By competing with v-FLIP, but not LC3, for binding to ATG3, the peptide mimics effectively reduced the size of the tumors [44]. Secondly, v-FLIP’s suppression of autophagy inhibits v-cyclin-induced cellular senescence during viral infection [45]. KSHV’s v-cyclin protein is an oncogenic mimic of the human cyclins that drives the proliferation of infected endothelial cells. When expressed alone in human foreskin fibroblasts (HFFs), v-cyclin induces senescence, but senescence is rarely observed during infection with KSHV [45]. Autophagy appears to contribute to cellular senescence in human fibroblasts, and its inhibition delays senescence in fibroblasts following the induction of the oncogene, Ras [11]. v-FLIP’s suppression of autophagy is both necessary and sufficient to inhibit senescence of KSHV-infected HFFs [45]. Thus, it appears that KSHV inhibits autophagy via v-FLIP and this inhibition contributes to the infected cells’ survival and potentially to its oncogenic progression. 

Another means by which KSHV can inhibit autophagy is through its ortholog of Bcl2 which binds Beclin 1 [29,37]. Bcl2 itself binds Beclin 1 only at the endoplasmic reticulum where the nutrient-deprivation autophagy factor 1 (NAF1) is localized [29,46]. NAF1 is a member of the inositol-1,4,5 triphosphate receptor complex and is required for Bcl2 to bind Beclin 1 [29]. In comparison to the human Bcl2, KSHV’s v-Bcl2 binds more strongly to Beclin 1, likely indicating a selection for this interaction in the life-cycle of KSHV [12]. KSHV’s v-Bcl2 is expressed during the productive phase of the viral life cycle and it is not clear what advantage this inhibition provides the virus. Inhibiting autophagy could limit the presentation of antigens on MHC class II molecules and thereby protect infected cells from their immune recognition [21,22]. It is all reasonable to speculate that KSHV’s v-Bcl2 is expressed soon after infection of B-cells or endothelial cells and blocks apoptosis as do the v-Bcl2 orthologs of EBV [27]. All of these viral orthologs may contribute to successful infections by their respective viruses by binding Beclin 1 to regulate autophagy, too. 

One reason the role of KSHV’s v-Bcl2 in inhibiting autophagy is uncertain is because the immediate-early transcription factor of KSHV, replication and transcription activator (RTA) stimulates autophagy, which in turn supports the productive phase of viral infection (Table 2, Table 3). The expression of RTA is necessary to induce the productive cycle of KSHV. For example, sodium butyrate stimulates the expression of RTA in latently infected cells and activates a transcriptional cascade of viral genes required for producing infectious virions [38,47]. Independently of KSHV, RTA can induce autophagy in endothelial cells and B-cells by an unknown mechanism [40]. Treating PEL cells with 3-methyl adenine (3-MA), an inhibitor of autophagy, represses expression of viral genes needed for viral replication [40]. KSHV’s productive cycle therefore benefits from autophagy. It is possible, given its inhibition of autophagy, that v-FLIP maintains viral latency, that is, blocks the transition from latent to productive infection via its inhibiting autophagy. KSHV uses autophagy to promote its productive cycle and inhibits it probably to foster its latent infections. Both activities would potentially contribute to the pathogenesis by KSHV. 

## 4. Hepatitis C Virus

Hepatitis C Virus (HCV) causes a large fraction of cases of primary hepatocellular carcinoma (HCC) in the world today with about 200 million people currently being infected [38]. The virus is a member of the Flavivirus family with its particle containing a single, positive-sense RNA genome of close to 10 kb in length [38]. The RNA is translated during infection into a single polypeptide that is subsequently cleaved by cellular and viral proteases into four structural and six non-structural proteins [38]. By “structural” proteins is meant those that make up the virus particle; non-structural proteins are usually enzymes that regulate the viral life-cycle. 

HCV infection initiated either by treatment of cells with infectious particles or transfection of viral RNAs into them induces autophagy (Table 2) [48,49]. It has also been found that HCV’s non-structural protein 4B, NS4B, when expressed alone in cells induces autophagy (Table 3) [39]. Multiple studies indicate that the inhibition of this induced autophagy inhibits HCV infection. The inhibition of Beclin 1 and ATG4B with siRNAs in infected Huh7 cells decreases the levels of synthesized viral RNA by 100-fold [48]. Beclin 1 is a major regulator of autophagy and ATG4B cleaves pro-LC3, generating the cytosolic LC3-I protein prior to its conjugation to PE [1,13]. NS5B has been found to bind ATG5 in yeast two-hybrid assays; inhibition of ATG5 with siRNAs in infected Huh7 cells inhibits viral RNA synthesis 10-fold (Table 3) [50]. ATG5 forms a complex with ATG12 and then with ATG16L, which together determine where in phagophores LC3 is conjugated to PE [4,51]. ATG5 interacts with immunity related GTPase M (IRGM) and the inhibition of IRGM with siRNA inhibits HCV infection, too [14].

The mechanism by which inhibiting autophagy inhibits HCV infection is being elucidated in order to understand what this virus gains by its inducing autophagy. Inhibiting the induction of autophagy prior to infection inhibits viral RNA synthesis, but it does not do so after HCV replication has been initiated [48]. This timing of the importance of autophagy in the HCV life-cycle is consistent with observations indicating that autophagy inhibits an innate immune response to HCV early in the viral life-cycle [49]. These authors found that the inhibition of ATG5 (or of participants in the UPR which foster autophagy) with siRNAs enhanced both transcription of the interferon beta (IFNβ) promoter and of downstream response elements, such as interferon-stimulated gene 56 (ISG56). Their findings are consistent with HCV-induced autophagy limiting the innate immune response of retinoic acid inducible gene I protein (RIG-I) recognizing sequences in HCV’s RNA genome [41]. This inhibition would be required by the virus only early during the course of infection because later in infection a viral protease, NS3/4A, cleaves the cellular protein, mitochondrial antiviral signaling protein (MAVS), which is required downstream of RIG-I to induce IFNα and β [52]. It now appears that HCV induces autophagy to block an interferon response and allow the early stages of its infection. HCV uses an independent mechanism to block this innate immune response later in its life-cycle. Inhibiting autophagy late in HCV’s life-cycle when it has established a persistent infection is detrimental to the virus because it induces death [53]. It therefore appears that even in persistently infected cells, HCV’s induction of autophagy is advantageous to the virus. 

## 5. Hepatitis B Virus

Three hundred and fifty million people worldwide are infected with Hepatitis B Virus (HBV) despite the availability of a vaccine that prevents infection. HBV causes a large fraction of cases of HCC, as does HCV. HBV is a member of the Hepadnavirus family and its linear, partially double-stranded DNA molecule of 3.2 kb is found in a protein core, which itself is enveloped [54]. The DNA is transcribed into a 3.4 kb pregenomic mRNA during infection, which is encapsidated by the viral core protein and serves as a template for reverse transcription [54]. This large mRNA, as well as smaller ones with the same poly-adenylation site, encodes three envelope surface proteins, a core protein, reverse transcriptase, and the X protein (HBx) [54]. The surface proteins have an identical, four-spanning transmembrane domain and the small surface protein, HBs can self-oligomerize.

Two groups have found that HBV induces autophagy in cells transfected *in vitro* and in mice transgenic for HBV (Table 2) [55,56,57]. Their combined data support this induction by HBV persuasively [55,56,57]. For example, transfection of cells derived from human liver tumors with HBV DNA induces increased levels of PE-conjugated LC3 [55] and the formation of autophagosomes [57]. These combined studies also show that autophagy fosters HBV’s life-cycle. Inhibiting autophagy with siRNAs to ATG7 inhibits formation of the products of HBV’s reverse transcription [55] and siRNAs to Beclin 1 inhibit the appearance of viral DNA in extracellular medium [57]. These findings are also supported by an analysis in mice engineered to be null for ATG5 in their livers [51]. The null mice when transgenic for HBV have greatly reduced levels of viral DNA in their sera when compared to their ATG5-positive counterparts [56]. These studies support the ability of HBV to induce autophagy and for the viral life-cycle to be fostered by autophagy. Where they differ is in their notions of the mechanisms of induction and the benefits that subsequently accrue to HBV. One set of experiments indicates that the viral HBx gene is essential for the induction of autophagy (Table 2) [55]; the other indicates that the HBs gene is sufficient for this induction (Table 2) [57]. One finds that the inhibition of autophagy inhibits reverse transcription within the viral core [55]; the other that the inhibition of autophagy inhibits viral envelopment [57]. These varied findings cannot be straightforwardly reconciled. It is apparent that expression of HBs in cells does induce the UPR and that the UPR leads to autophagy in these cells [57]. Inhibiting key mediators of the UPR such as PERK or ATF6 with siRNAs does block autophagy in cells transfected with HBs, for example [51]. Perhaps some of the differences noted in the studies reflect differences in the authors’ emphasis more than in their science. HBV likely induces and benefits from autophagy; the specifics of how it does so need elaboration. 

## 6. Discussion

**Figure 1:**
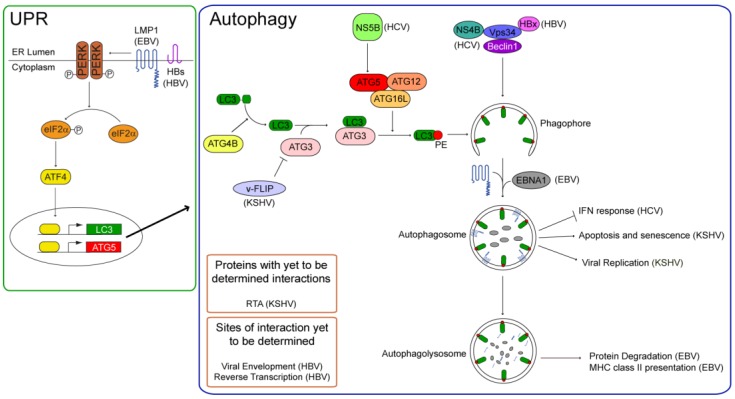
Autophagy is initiated by stimuli that activate the phosphatidylinositol 3-phosphate kinase, Vps34. Vps34 generates phosphatidylinositol 3-phosphate, a substrate for the forming autophagosome, and its enzymatic activity is dependent on Beclin 1. For its localization in the phagophore, the unclosed double-membrane precursor to the autophagosome, the light chain 3 (LC3) is conjugated to phosphatidylethanolamine (PE) via an ubiquitin-like conjugation pathway. LC3 is cleaved from its inactive precursor form by ATG4B to render it cytosolic, and subsequently conjugated to PE via its binding the E2-like ligase, autophagy related 3 homolog (ATG3), and its transfer to the E3-like ATG16L complex (ATG16L, ATG5, and ATG12). Autophagy can be stimulated via the Unfolded Protein Response (UPR) via the phosphorylation and activation of protein kinase RNA-like endoplasmic reticulum kinase (PERK). Active PERK phosphorylates the eukaryotic initiation factor 2 alpha (eIF2α). Phospho-eIF2α cannot translate capped mRNAs, but can translate mRNAs with internal ribosome entry sites (IRES), such as activated transcription factor 4 (ATF4). ATF4 activates the transcription of LC3 and Beclin 1 to induce autophagy. Human tumor viruses can regulate autophagy at several steps of the pathway. Both latent membrane protein 1 (LMP1) and the small surface protein of HBV (HBs) stimulate the UPR and induce autophagy. LMP1’s induction of autophagy regulates its degradation in autophagolysosomes. The Epstein-Barr nuclear antigen 1 (EBNA1) also can localize to autophagosomes and is degraded in autophagolysosomes, where epitopes of EBNA1 are generated for their presentation on MHC class II molecules. Autophagy fosters HBV’s productive infection. In addition to HBs, the Hepatitis B X protein (HBx) can stimulate autophagy via its interaction with Vps34. Autophagy can support HBV’s envelopment or reverse transcription. The non-structural proteins of HCV, NS4B and NS5B induce autophagy; the former appears to interact with Vps34, while the latter promotes LC3’s conjugation to PE by binding ATG5. Autophagy inhibits the cellular interferon response during the early stages of HCV’s infection. In contrast to NS5B, the KSHV viral FADD-like interleukin-1 beta-converting enzyme inhibitory protein (v-FLIP) inhibits LC3’s lipid-conjugation by competing with it for ATG3. v-FLIP inhibits autophagy-induced apoptosis and senescence of KSHV-positive cells. Autophagy is induced by replication and transactivator (RTA) to support KSHV’s replication, and v-FLIP may inhibit the productive life-cycle of the virus by repressing autophagy.

Some viruses that interact with the autophagic machinery repress it, thus ensuring their success in infected cells. The four human tumor viruses reviewed here all use—and even stimulate—autophagy for their own benefits (Figure 1). Each virus encodes one or more proteins that regulate the autophagic machinery either directly or indirectly.

All four human tumor viruses induce autophagy, and KSHV also inhibits it. Two of these viruses, HBV and EBV, encode transmembrane proteins—HBs and LMP1, respectively—that localize to the endoplasmic reticulum and induce autophagy via the UPR [35,57]. In its induction of autophagy in EBV-positive B-cells, the viral protein LMP1 autocatalyzes its degradation, thereby likely avoiding immune detection and relieving the cytostasis it causes [34]. How LMP1 and HBs activate the UPR is not known. It is possible that their complex membrane structures are easily misfolded. It is also possible that via their self-oligomerization they are recognized as protein aggregates that require degradation. Evidence consistent with the latter idea comes from models of Parkinson’s disease [58]. 

Whereas LMP1’s induction of autophagy may contribute to EBV’s avoidance of immune detection, the EBNA1 protein encoded by EBV can be presented on MHC class II molecules in part by autophagy [6,23]. HCV also uses autophagy to avoid immune detection. During the early stages of infection, NS5B of HCV interacts with ATG5 and stimulates the conjugation of LC3 to PE [50]. Autophagy inhibits the IFN response stimulated by RIG-I when liver cells are infected with HCV [49,53]. Though autophagy’s inhibition of innate immunity is required only for early infection, autophagy appears also to inhibit cellular death during the late stages of HCV’s infection. 

Autophagy fosters the productive life-cycles of KSHV, HBV, and HCV; more virions are produced when autophagy is induced by viral proteins [40,48,55,56,57,59]. KSHV can also inhibit autophagy. Its v-FLIP, which is expressed during viral latency, competes with LC3 for binding to ATG3, thereby inhibiting LC3’s lipid-conjugation [44]. In doing so, v-FLIP inhibits cellular death of KSHV-infected PEL cells and HFFs [44]. KSHV’s v-Bcl2 can also inhibit autophagy by binding Beclin1 but the role this inhibition plays in the viral life-cycle is now unknown. 

Given its role in the life-cycle of HCV, HBV, KSHV, and EBV, autophagy could be a potential target for anti-viral or tumor therapy. For example, suppressing autophagy would block the production of HBV and HCV virions; it could cause cellular death in EBV- and HCV-positive cells. In contrast, cellular death of KSHV-positive PELs and Kaposi’s Sarcomas could occur when autophagy is induced. Elucidating the roles of autophagy in the life-cycles of human tumor viruses may lead to the identification of targets for new treatments of the many cancers these viruses cause. 
